# Development and validation of a scale for the psychological determinants of dietary management behavior in hemodialysis patients during dialysis

**DOI:** 10.1371/journal.pone.0351672

**Published:** 2026-06-11

**Authors:** Taofeng Wu, Yingying Jiang, Hongyun Yan, Jingfang Chen, Xianrong Xu, Xingxing Shen

**Affiliations:** 1 The Affiliated Suzhou Hospital of Nanjing Medical University, Suzhou Municipal Hospital, Gusu School of Nanjing Medical University, China; 2 Jiangsu Provincial People's Hospital Hemodialysis Center, China; 3 Department of Medical Nursing, Suzhou Vocational Health College, China; Tribhuvan University Institute of Medicine, NEPAL

## Abstract

**Objective:**

This study aimed to develop and validate a scale for psychological determinants and behavioral performance of diet management behaviors in hemodialysis patients during dialysis sessions.

**Methods:**

A preliminary item pool was generated based on the theory of planned behavior and evidence synthesis. The Delphi method, involving 19 experts, was employed to refine the scale content, resulting in an initial draft. Participants were recruited from four hemodialysis centers in Suzhou between 01/04/2024 and 31/05/2024. This draft was then administered to 418 hemodialysis patients from these centers. Data were analyzed using SPSS 25.0 and AMOS 13.0 for item analysis, reliability, and validity assessment.

**Results:**

The response rate across two Delphi rounds was 100%. The expert authority coefficient was 0.88, and Kendall’s concordance coefficient for the second round was 0.245 (P < 0.05). The final scale comprised three dimensions (attitude, subjective norms, and perceived behavioral control) with 21 items. The overall Cronbach’s α was 0.964, with subscale α coefficients ranging from 0.918 to 0.938. Split-half reliability was 0.914, and test-retest reliability was 0.657. The scale-level content validity index (S-CVI) was 0.86, and item-level CVI (I-CVI) ranged from 0.800 to 1.000. Exploratory factor analysis extracted three common factors, accounting for 77.447% of the cumulative variance. Confirmatory factor analysis showed an acceptable marginal model fit after residual error modification.

**Conclusion:**

The developed Diet Management Behavior Scale for Hemodialysis Patients demonstrates acceptable reliability and validity. Given the limitations of regional sampling and moderate expert consensus, the scale is preliminarily suitable for assessing psychological determinants of diet management and behavioral status during dialysis.

## Introduction

The prevalence of chronic kidney disease (CKD) among adults in China is approximately 10.8%, with the number of end-stage renal disease (ESRD) patients increasing at an annual rate of nearly 7% [1,2]. Among these patients, about 89% undergo hemodialysis as their primary treatment [3]. Maintaining appropriate dietary intake during hemodialysis sessions is recognized as a measure to reduce hunger and hypoglycemia [4,5], improve nutritional status [6–8], and enhance patient-provider relationships [9]. However, maintenance hemodialysis (MHD) patients often experience adverse clinical symptoms such as hypotension and indigestion after eating during dialysis [10], which can directly compromise dialysis adequacy and treatment efficacy. Therefore, effective diet management during dialysis is crucial for MHD patients, and accurately assessing their dietary behavior and its psychological influencing factors during this period is essential for implementing targeted nutritional interventions.

Existing scales for assessing dietary behaviors in hemodialysis patients primarily focus on overall self-management and lack specificity and granularity in evaluating nutritional practices [11–14]. Moreover, these instruments are generally designed to assess dietary management at home, with none specifically developed to evaluate diet management during dialysis sessions. The Theory of Planned Behavior (TPB) indicates that attitude, subjective norms, and perceived behavioral control are core psychological antecedents of behavioral intention and further predict individual actual behavior [15]. Under this theoretical framework, assessing psychological cognition and behavioral performance of diet management during dialysis serves as the foundation for designing behavioral interventions.

Accordingly, this study aims to develop and validate a scale to assess diet management behaviors and their psychological influencing factors during dialysis in MHD patients. The resulting instrument is intended to provide clinical nursing staff with a scientifically sound and practical tool to evaluate patients’ dietary management cognition and behavioral intention during hemodialysis, facilitate a better understanding of their real-time nutritional behavior, and support the design of tailored dietary interventions to potentially improve dialysis outcomes and quality of life.

## Methods

### Formation of the research team

The research team consisted of 12 members with backgrounds in medicine and statistics, all of whom had completed relevant training. The team’s core responsibilities included constructing the item pool, designing and implementing the expert consultation process, summarizing and analyzing expert feedback to adjust item content, and performing data analysis.

### Theoretical framework

The TPB theory suggests that attitudes, subjective norms, and perceived behavioral control collectively form the psychological intention of individual behavior, and further explain and predict personal actual behavior. This theory has been widely applied in various fields. By examining these three TPB dimensions, this study explores hemodialysis patients’ cognitive and emotional evaluation of diet management during dialysis, investigates the influence of perceived social pressure and others’ expectations on their dietary management behavioral intention, and assesses patients’ confidence in controlling dietary behaviors and their perception of external factors affecting these behaviors. Based on the TPB theory, the structure of the scale was determined, and the item pool was formed.

### Preliminary formation of the item pool based on best evidence

The research team conducted a comprehensive literature review following the evidence pyramid model [16], searching databases such as UpToDate, the Scottish Intercollegiate Guidelines Network, the National Guideline Clearinghouse, the National Institute for Health and Care Excellence (NICE), the Registered Nurses Association of Ontario (RNAO), the Chinese Clinical Guideline Library, the Joanna Briggs Institute (JBI) Evidence-Based Healthcare Center Library, Cochrane Library, PubMed, Embase, Medline, the Chinese Biomedical Literature Database, CNKI, and Wanfang Data. Keywords included “chronic kidney disease/hemodialysis/HD/maintenance hemodialysis,” “diet/eating/food intake/nutrition/meals,” and “dialysis-related hypotension/intradialytic hypotension/dialysis adequacy/nutritional indicators/gastrointestinal reactions/vomiting/nausea/choking/dialysis complications/adverse events during dialysis/blood glucose.” A total of 1,038 articles were initially retrieved, and after excluding irrelevant and duplicate articles, 37 articles were included. After reading and quality evaluation, eight articles were finally included [17–24], comprising two guidelines, two expert consensus documents, three clinical decision-making articles, and one systematic review. These were integrated into eight best evidence statements for diet management during dialysis in MHD patients [25]. Based on this evidence, the research team formed the initial item pool.

### Expert focus group interviews to supplement the item pool

One nephrologist, two chief nurses from hemodialysis centers, and two deputy chief nurses from hemodialysis centers were invited to discuss the item pool formed from the best evidence analysis. After discussion, the initial draft of the scale was formed, consisting of three dimensions: attitude (7 items), subjective norms (7 items), and perceived behavioral control (5 items), totaling 19 items.

### Expert consultation

The Delphi method was used for expert consultation with 19 multidisciplinary experts. Two rounds of expert consultation questionnaires were distributed via email or chat software. The first-round questionnaire included three parts: (1) a letter to the experts briefly explaining the research background, purpose, methods, and instructions for completing the questionnaire; (2) the initial questionnaire on diet management during dialysis in MHD patients, including item content, importance and relevance ratings, and space for comments and suggestions for adding or deleting items; and (3) expert information, including general information, judgment basis, and familiarity with the survey content. The second-round questionnaire included (1) a letter to the experts and a summary of the first-round expert opinions and modifications, and (2) the revised scale. Expert selection criteria included: (1) working in hemodialysis, nursing education, nursing management, or nutrition for at least 10 years; (2) holding a bachelor’s degree or higher; (3) having an intermediate or higher professional title; and (4) agreeing to participate in the study. Items were evaluated for importance and relevance using a 5-point Likert scale, with scores ranging from “very unimportant” to “very important” and “very irrelevant” to “very relevant.” Experts were asked to provide comments and suggestions for modifying or adding items. Items with an average score of less than 3.5 and a coefficient of variation greater than 0.25 were excluded [26]. After two rounds of expert consultation, the initial version of the scale was formed, consisting of 21 items.

### Pilot survey

A convenience sample of 30 MHD patients from a tertiary hospital was selected to complete the questionnaire formed after two rounds of expert consultation. The survey was conducted via QR code scanning to test the clarity and comprehensibility of the items. Participants were from different age groups, dialysis durations, and educational levels.

### Reliability and validity

#### Study population.

A cross-sectional survey was conducted using convenience sampling to select MHD patients from four hemodialysis centers in Suzhou between 01/04/2024 and 31/05/2024. Inclusion criteria were: (1) receiving regular hemodialysis treatment for at least 3 months; (2) aged 18 years or older; and (3) informed written consent was obtained from all participants. Exclusion criteria were: (1) severe complications such as severe infections or acute heart failure; (2) cognitive impairment, dementia, or mental illness; and (3) communication barriers preventing interaction with the researcher. According to scale design principles, exploratory factor analysis requires a sample size of 5–10 times the number of items [27].and confirmatory factor analysis requires a sample size of at least 200 [28]. Considering a 10% invalid response rate, the total sample size was calculated to be 339–456. All participants signed written informed consent.

This study was approved by the Institutional Review Board/Ethics Committee of Suzhou Municipal Hospital，Ethics Approval No.: K-2024–036-K01. All procedures performed in this study were in accordance with the ethical standards of the institutional research committee and with the 1964 Helsinki declaration and its later amendments. All participants provided voluntary written informed consent prior to enrollment.

### Study tools

**General information questionnaire**, including gender, age, dialysis duration, body mass index (BMI), vascular access, type of medical insurance, and caregiver status; **The Diet Management Behavior Scale for Hemodialysis Patients during Dialysis,** consisting of three dimensions (attitude, subjective norms, and perceived behavioral control) and 21 items, all rated on a 5-point Likert scale, with higher scores indicating better behavioral tendency and management performance.

### Item analysis

Four methods were used for item analysis: critical ratio method, item-total correlation, Cronbach’s α coefficient, and commonality and factor loading tests [29]. The criteria were: (1) independent samples t-test for the top 27% and bottom 27% of total scores, with items showing no significant difference excluded; (2) items with a correlation coefficient of less than 0.400 with the total score excluded; (3) items whose removal significantly increased the Cronbach’s α coefficient excluded; and (4) items with a commonality value of less than 0.2 or a factor loading of less than 0.45 excluded.

### Validity testing

**Content Validity:** Ten hemodialysis nursing professionals were invited to rate the content validity of the questionnaire. The content validity index (CVI) for each item (I-CVI) and the scale-level content validity index (S-CVI) were calculated, with I-CVI > 0.780 and S-CVI ≥ 0.800 considered acceptable [30].

**Construct Validity:** The total sample was randomly divided into two groups using a random number generator, with sample 1 used for exploratory factor analysis and sample 2 for confirmatory factor analysis. Exploratory factor analysis was conducted with the following criteria: cumulative variance contribution rate ≥ 50%, factor loading ≥ 0.400, and eigenvalue > 1. Items with dual loadings greater than 0.400 and a difference of less than 0.200 were excluded. ^30^ Confirmatory factor analysis was conducted with the following criteria: χ²/df < 3; comparative fit index (CFI), goodness-of-fit index (GFI), normed fit index (NFI), Tucker-Lewis index (TLI), and incremental fit index (IFI) > 0.900; root mean square error of approximation (RMSEA) < 0.080; and root mean square residual (RMR) < 0.05 [30].

### Reliability testing

Reliability was assessed using three methods: ① Cronbach’s α coefficient: a value greater than 0.700 was considered acceptable for overall scale reliability [31]; ② split-half reliability: the scale items were divided into two halves (odd and even), with a split-half reliability coefficient above 0.800 considered stable; ③ test-retest reliability: 30 patients were retested after two weeks, and the correlation between the two scores was calculated to assess the scale’s test-retest reliability. The moderate test-retest reliability (0.657) was interpreted as borderline acceptable, which is inconsistent with high internal consistency, possibly related to short retest interval and patients’ transient dietary cognition changes.

### Data collection

Data were collected both online and offline. Online data collection was conducted via QR code scanning, while offline data were collected through one-on-one interviews. Before the survey, researchers explained the study purpose and content in detail, ensured informed consent, and maintained confidentiality. The estimated time to complete the questionnaire was 5–10 minutes. Quality control measures included limiting responses to one per participant and requiring all questions to be answered before submission. Invalid questionnaires with obvious patterns were excluded.

### Data analysis

Data were analyzed using AMOS 13.0 and IBM SPSS Statistics 25.0. Expert consultation reliability was assessed using familiarity, authority coefficient, and Kendall’s concordance coefficient. Normally distributed continuous data were described using mean and standard deviation, while categorical data were described using frequency and percentage. Exploratory factor analysis, confirmatory factor analysis, and internal consistency testing were used to assess the scale’s validity. Cronbach’s α coefficient was used to assess the scale’s reliability. A P-value < 0.05 was considered statistically significant.

## Results

### Expert consultation results

Nineteen experts from six cities, including Nanjing, Suzhou, Wuxi, and Hefei, were selected. The experts included seven hemodialysis nursing specialists, ten nursing management experts in hemodialysis, one nursing education expert, and one nephrologist. The experts were aged 35–57 years, with 10–35 years of work experience and 1–25 years of experience in hemodialysis. Fourteen experts held senior professional titles, and five held intermediate titles. Four experts held master’s degrees or higher, and 15 held bachelor’s degrees. Two rounds of expert consultation were conducted, with a 100% response rate in both rounds, indicating high expert engagement. Fourteen experts (73.68%) provided feedback in the first round, and two experts (10.52%) provided feedback in the second round. The Kendall’s concordance coefficient for item importance was 0.183 in the first round and 0.245 in the second round, while the coefficient for item relevance was 0.192 in the first round and 0.244 in the second round. After two rounds of consultation, all 21 items had an average score above 3.5 and a coefficient of variation below 0.25. The average importance score for each item ranged from 4.00 to 5.00, with a coefficient of variation (CV) of 0-0.204 and a full score ratio of 0.31–1.00. The average relevance score for each item ranged from 4.26 to 5.00, with a CV of 0.000−0.172. Two items were added to the attitude dimension: “I believe in the professionalism of medical staff in managing diet during hemodialysis“ and “I am confident that proper diet management during dialysis can enhance my confidence in disease control.“ One item was removed from the subjective norms dimension: “I feel that the medical team strongly expects me to actively participate in good diet management during dialysis.“ One item was added to the perceived behavioral control dimension: “I believe I can effectively implement the medical team’s dietary recommendations during dialysis.“ Two items in the attitude dimension were modified, one item in the subjective norms dimension was modified, and one item in the perceived behavioral control dimension was modified. After the second round of consultation, expert opinions converged, and the initial version of the scale was formed, consisting of three dimensions and 21 items. The scale used a 5-point Likert scale, with scores ranging from 1 (“strongly disagree”) to 5 (“strongly 6-agree“).

### Pilot survey results

Thirty questionnaires were distributed for the pilot survey, and all were returned. Participants found the language of the items clear and understandable, and the time to complete the questionnaire was less than 10 minutes. No additional items or modifications were suggested.

#### General information of survey participants.

A total of 426 questionnaires were collected, and after excluding four questionnaires with completion times of less than 5 minutes, 418 valid questionnaires were obtained, with a valid response rate of 98.12%. The general information of the 418 patients is shown in Table 1.

**Table 1:** General Information of Patients.

Item		Value (Mean ± SD) / Percentage (%)
Gender	Male	248（59.3%）
	Female	170（40.7%）
Age (years)		62.03 ± 14.20
Dialysis Duration (years)		6.50 ± 6.79
BMI		22.36 ± 3.64
Vascular Access	Arteriovenous fistula	362（86.6%）
	Central venous catheter	34（8.1%）
	Both	22（5.3%）
Type of Medical Insurance	Medical insurance	381（91.1%）
	Agricultural insurance	31（7.4%）
	Self-pay	4（1.0%）
	Other	2（0.5%）
Education Level	Illiterate	32（7.7%）
	Junior high school or belo	210（50.2%）
	High school	144（34.4%）
	Bachelor’s degree	32（7.7%）
Living Situation	Living alone	70（16.7%）
	Not living alone	348（83.3%）
Marital Status	Unmarried	30（7.2%）
	Married	335（80.1%）
	Divorced	26（6.2%）
	Widowed	27（6.5%）
Caregiver Status	No caregiver	74（17.7%）
	Parent caregiver	32（7.7%）
	Child caregiver	186（44.5%）
	Hired caregive	10（2.4%）
	Other	116（27.7%）
Monthly Household Income (CNY)	< 3000	117（28.0%）
	3000-5000	159（38.0%）
	> 5000	142（34.0%）
Primary Disease	Chronic nephritis	94（22.5%）
	Glomerulonephritis	24（5.8%）
	Diabetic nephropathy	74（17.7%）
	Hypertensive nephropathy	140（33.5%）
	Polycystic kidney disease	34（8.1%）
	Systemic lupus erythematosus	6（1.4%）
	Other	46（11.0%）
Comorbidities	Hypertension	343（82.1%）
	Diabetes	94（22.5%）
	Coronary heart disease	54（12.9%）
	Renal osteodystrophy	37（8.9%）
	Other	44（10.5%）
Dietary Education during Dialysis	Received	177（42.3%）
	Not received	241（57.7%）

### Content validity results

The content validity index (CVI) for each item ranged from 0.8 to 1. The CVI for the attitude, subjective norms, and perceived behavioral control dimensions were 0.89, 0.80, and 0.83, respectively. The overall CVI for the scale was 0.86.

### Construct validity results

Exploratory factor analysis was conducted on sample 1 (n = 209). The Kaiser-Meyer-Olkin (KMO) value was 0.938, and Bartlett’s test of sphericity was significant (χ² = 5040.959, P < 0.001), indicating suitability for factor analysis. Three common factors were extracted, with all items having factor loadings greater than 0.400. The cumulative variance contribution rate was 77.447%. The final scale consisted of three dimensions: attitude (9 items), subjective norms (6 items), and perceived behavioral control (6 items), totaling 21 items. The factor loading matrix is shown in Table 2.

**Table 2:** Factor Loading Matrix of the Diet Management Behavior Scale for Hemodialysis Patients.

Dimension	Item	Attitude	Perceived Behavioral Control	Subjective Norms
**Attitude**	I firmly believe that proper dietary management during dialysis can improve the effectiveness of dialysis treatment	**0.768**	0.280	0.382
	I trust the professionalism of medical staff in dietary management knowledge during hemodialysis	**0.764**	0.356	0.208
	I firmly believe that reasonable dietary management during hemodialysis is one of the key factors for maintaining safety	**0.764**	0.311	0.354
	Implementing reasonable dietary management during dialysis helps reduce discomfort during the dialysis process	**0.745**	0.311	0.360
	I deeply recognize that good dietary management during hemodialysis treatment is crucial for reducing adverse reactions to dialysis	**0.744**	0.311	0.305
	I firmly believe that proper dietary management during dialysis can enhance my confidence in disease control	**0.733**	0.372	0.335
	Dietary management during dialysis can alleviate anxiety and tension	**0.732**	0.288	0.280
	Implementing a reasonable dietary management plan during dialysis gives me a sense of security and benefit	**0.697**	0.212	0.207
	I believe that persistent dietary management during dialysis can improve my quality of life	**0.681**	0.350	0.445
**Perceived Behavioral Control**	I can strictly follow the advice of medical staff regarding eating during dialysis	0.190	**0.855**	0.228
	I have the ability to judge whether my physical condition is suitable for eating during dialysis and strictly implement this judgment	0.300	**0.827**	0.303
	I can clearly understand the types of food, food quantity, eating methods, etc., recommended by medical staff	0.345	**0.785**	0.313
	I have the basic ability to judge food nutrients and reasonably arrange my diet	0.385	**0.783**	0.278
	I have sufficient ability and time to prepare food that meets the medical staff’s recommendations	0.396	**0.776**	0.274
	Even when facing difficulties during dialysis treatment, I can find ways to adhere to dietary management	0.314	**0.646**	0.143
**Subjective Norms**	The strong expectations of the medical team positively influence my active participation in proper diet management during dialysis	0.381	0.203	**0.796**
	The good dietary behaviors of fellow patients encourage me to follow dietary recommendations during dialysis	0.392	0.230	**0.794**
	The support of family and friends helps me better follow dietary advice during hemodialysis	0.322	0.362	**0.791**
	The expectations of family and friends encourage me to better follow dietary advice during hemodialysis	0.418	0.254	**0.759**
	The dietary education provided by the medical team has a positive impact on my active participation in good dietary management during hemodialysis	0.463	0.177	**0.751**
	Advice on dietary management during dialysis from social media (WeChat APP, Internet, popular science videos, etc.) has a positive impact on me	0.110	0.351	**0.720**
**Eigenvalue**		**6.338**	**4.994**	**4.931**
**Variance Contribution Rate**		**30.182%**	**23.783%**	**23.482%**
**Cumulative Variance Contribution Rate**		**30.182%**	**53.965%**	**77.447%**

### Confirmatory factor analysis results

**Fig 1:**
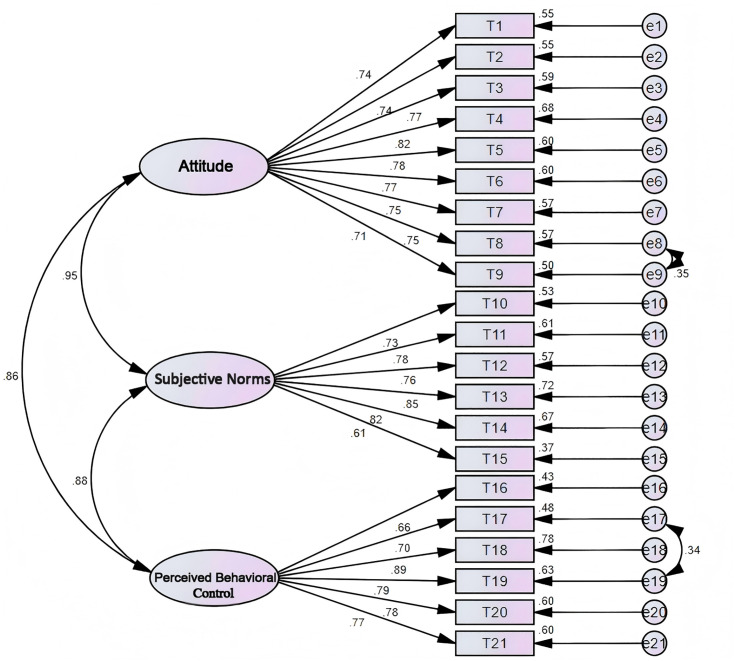
Confirmatory Factor Analysis Structural Model of the Diet Management Behavior Scale for Hemodialysis Patients.

Confirmatory factor analysis was conducted on sample 2 (n = 209). The initial model fit was poor, and modifications were necessary based on the theoretical connotation similarity of items. After establishing a covariance relationship between the residuals of items T8 and T9, the fit indices improved, but the model still did not fit well. After a second modification of the residuals of items T17 and T19, the confirmatory factor analysis results showed: χ²/df = 2.276, GFI = 0.849, CFI = 0.927, NFI = 0.877, TLI = 0.916, IFI = 0.927, RMR = 0.022, and RMSEA = 0.078, GFI and NFI were slightly lower than the conventional 0.90 threshold, and the model showed a marginally acceptable fitting level. The structural model of the confirmatory factor analysis is shown in Fig 1. The correlation of residuals between items T8–T9 and T17–T19 was not purely data-driven modification, but based on theoretical connotation and measurement homogeneity of scale items. Specifically, items T8 and T9 both belong to the attitude dimension, focusing on patients’ cognitive evaluation and emotional recognition of the necessity and benefit of dietary management during dialysis; the two items share highly similar measurement scope, implicit logical correlation and overlapping situational background, resulting in inherent residual association. Similarly, items T17 and T19 are included in the perceived behavioral control dimension, both involving patients’ self-perception of behavioral difficulty, external obstacle interference and self-efficacy in implementing dietary dietary management recommendations during dialysis. The two items measure homologous behavioral control perception under similar clinical scenarios, which has rational theoretical and substantive content correlation. Therefore, allowing residual covariance between the paired item sets is consistent with the connotation design of the planned behavior theory and the scale dimension framework, rather than arbitrary modification only for improving model fit.

### Reliability analysis results

The overall Cronbach’s α coefficient for the scale was 0.964, indicating excellent internal consistency, while such a high value may imply potential item redundancy and content overlap among scale entries. The Cronbach’s α coefficients for the three dimensions ranged from 0.918 to 0.938. The split-half reliability was 0.914, and the test-retest reliability was 0.657,showing only moderate temporal stability and inconsistent with high internal consistency. Inter-item correlation analysis further showed that most correlation coefficients between individual items ranged from 0.612 to 0.856, with only a small number of item pairs exceeding 0.850, indicating moderate to high correlation among scale items. The high overall Cronbach’s α coefficient of 0.964 reflected excellent internal consistency of the whole scale, but also implied partial content overlap and potential item redundancy among several closely related items within the same dimension. No single item could be deleted obviously, without reducing the structural integrity and theoretical coverage of the three TPB dimensions. Therefore, we retained all 21 items to ensure complete theoretical framework and clinical comprehensiveness, and suggested that future research could appropriately simplify individual highly correlated items to optimize scale parsimony and discriminability.

## Discussion

**The Diet Management Behavior Scale for Hemodialysis Patients is Scientific and Reliable.** This study was based on a comprehensive literature review, forming a “Summary of Best Evidence for Diet Management during Dialysis in MHD Patients” [17], providing a solid theoretical foundation for the development of the scale. The scale was developed based on the Theory of Planned Behavior (TPB) and expert panel discussions, incorporating the opinions of medical professionals, making it highly relevant to clinical practice. The three dimensions of the scale assess patients’ cognition, emotions, and behaviors related to diet management during dialysis, providing a detailed understanding of patients’ dietary management behaviors and offering an effective tool for healthcare professionals to implement targeted interventions. The experts consulted in this study were from tertiary hospitals and universities in six cities, including Nanjing, Suzhou, Wuxi, and Hefei. All 19 experts had extensive clinical experience and theoretical knowledge, covering fields such as clinical medicine, clinical nursing, hemodialysis nursing, nursing management, and nursing education, making them highly representative. The response rate for the two rounds of expert consultation was 100%, with 73.68% and 10.52% of experts providing constructive feedback in the first and second rounds, respectively, indicating high expert engagement and interest. The expert authority coefficient was 0.88, indicating high credibility and reliability of the selected experts. The Kendall’s concordance coefficients were statistically significant but at a moderate level, suggesting only moderate consensus among experts, rather than strong opinion convergence.

**The Diet Management Behavior Scale for Hemodialysis Patients Demonstrates Good Reliability and Validity.** In terms of content validity, the scale-level content validity index (S-CVI) was 0.86 (> 0.800), and the item-level content validity index (I-CVI) ranged from 0.800 to 1.000 (> 0.780), indicating good content validity. Exploratory factor analysis revealed three common factors, with a cumulative variance contribution rate of 77.447%. All factor loadings were above 0.5, indicating acceptable construct validity. CFA modification was conducted on the basis of item theoretical similarity, and the final model achieved marginally acceptable fit, though GFI and NFI did not reach the ideal threshold.

The overall Cronbach’s α coefficient was 0.964 with high internal consistency, but the overly high value may reflect item redundancy; the moderate test-retest reliability (0.657) reflected limited long-term stability.

Limitations of the present study: First, convenience sampling was only adopted in four hemodialysis centers in Suzhou, with obvious regional homogeneity, which limits the external generalizability of the scale. Cross-regional and cross-cultural verification is required in future studies. Second, this study did not conduct criterion validity testing, including correlation analysis with existing dietary adherence scales and clinical indicators such as intradialytic hypotension, serum albumin and dialysis adequacy. Third, the scale cannot directly predict actual clinical outcomes, and the conclusion of improving dialysis outcomes cannot be inferred from current data. Fourth, the high Cronbach’s α suggests possible item overlap, and inter-item correlation and item deletion analysis should be further supplemented to optimize scale parsimony.

**The Diet Management Behavior Scale for Hemodialysis Patients Has Preliminary Clinical Practical Value.** The scale comprehensively assesses patients’ psychological cognition and behavioral intention of diet management during dialysis from the TPB＇s three dimensions. The “attitude” dimension helps quantify patients’ internal cognitive tendency toward diet management; the “perceived behavioral control” dimension evaluates patients’ self-perception of dietary implementation barriers; the “subjective norms” dimension reflects social and family environmental influence on behavioral intention. This scale can assist clinical staff in understanding patients’ dietary management cognition and intention, and formulate personalized guidance plans. However, its application effect on actual dialysis outcomes still needs further longitudinal verification.

## Conclusion

The Diet Management Behavior Scale for Hemodialysis Patients developed in this study consists of three dimensions and 21 items, with acceptable reliability, validity, and a rational theoretical framework. It can be used as a preliminary clinical assessment tool for evaluating psychological determinants and behavioral intention of dietary management in MHD patients during dialysis. Future research should conduct multicenter cross-regional sampling verification, supplement criterion validity and inter-item correlation analysis, optimize item settings, and explore influencing factors of dietary management behaviors, so as to further improve the scale’s generalizability and clinical application value.

### Practical application

Adverse clinical manifestations such as intradialytic hypotension and digestive discomfort following voluntary food intake in maintenance hemodialysis (MHD) patients can directly compromise dialysis adequacy and treatment outcomes. The diet management behavior scale developed in this study provides a validated tool for assessing dietary psychological intention and self-regulation tendency among hemodialysis patients during dialysis treatment. This targeted assessment facilitates personalized nutritional interventions to standardize dietary management and reduce dialysis-related adverse events, while its impact on long-term dialysis prognosis needs further clinical verification.

## Supporting information

S1 Data Dataset. (XLSX)
